# A Remote Patient Monitoring System With Feedback Mechanisms Using a Smartwatch: Concept, Implementation, and Evaluation Based on the activeDCM Randomized Controlled Trial

**DOI:** 10.2196/58441

**Published:** 2024-11-22

**Authors:** Reto Wettstein, Farbod Sedaghat-Hamedani, Oliver Heinze, Ali Amr, Christoph Reich, Theresa Betz, Elham Kayvanpour, Angela Merzweiler, Christopher Büsch, Isabell Mohr, Birgit Friedmann-Bette, Norbert Frey, Martin Dugas, Benjamin Meder

**Affiliations:** 1 Institute of Medical Informatics Heidelberg University Hospital Heidelberg Germany; 2 Institute for Cardiomyopathies Heidelberg Heidelberg University Hospital Heidelberg Germany; 3 German Centre for Cardiovascular Research Heidelberg-Mannheim Germany; 4 Department of Internal Medicine III Heidelberg University Hospital Heidelberg Germany; 5 RheinMain University of Applied Sciences Wiesbaden Germany; 6 Department of Sports Medicine Medical Clinic Heidelberg University Hospital Heidelberg Germany; 7 Institute of Medical Biometry Heidelberg University Heidelberg Germany

**Keywords:** wearable, consumer device, mobile phone, mobile health, telemedicine, remote patient monitoring, usability, Health Level 7 Fast Healthcare Interoperability Resources, HL7 FHIR, cardiology, heart failure, dilated cardiomyopathy

## Abstract

**Background:**

Technological advances allow for recording and sharing health-related data in a patient-centric way using smartphones and wearables. Secure sharing of such patient-generated data with physicians would enable close management of individual health trajectories, monitoring of risk factors, and asynchronous feedback. However, most remote patient monitoring (RPM) systems currently available are not fully integrated into hospital IT systems or lack a patient-centric design.

**Objective:**

The objective of this study was to conceptualize and implement a user-friendly, reusable, interoperable, and secure RPM system incorporating asynchronous feedback mechanisms using a broadly available consumer wearable (Apple Watch). In addition, this study sought to evaluate factors influencing patient acceptance of such systems.

**Methods:**

The RPM system requirements were established through focus group sessions. Subsequently, a system concept was designed and implemented using an iterative approach ensuring technical feasibility from the beginning. To assess clinical feasibility, the system was used as part of the activeDCM prospective randomized interventional study focusing on dilated cardiomyopathy. Each patient used the system for at least 12 months. The System Usability Scale was used to measure usability from a subjective patient perspective. In addition, an evaluation was conducted on the objective wearable interaction frequency as well as the completeness of transmitted data classified into sensor-based health data (SHD) and patient-reported outcome measures (PROMs). Descriptive statistics using box plots and bootstrapped multiple linear regression with 95% CIs were used for evaluation analyzing the influence of age, sex, device experience, and intervention group membership.

**Results:**

The RPM system comprised 4 interoperable components: patient devices, a data server, a data viewer, and a notification service. The system was evaluated with 95 consecutive patients with dilated cardiomyopathy (28/95, 29% female; mean age 50, SD 12 y) who completed the activeDCM study protocol. The system’s app achieved a mean System Usability Scale score of 78 (SD 17), which was most influenced by device experience. In total, 87% (83/95) of the patients could integrate the use of the app well or very well into their daily routine, and 71% (67/95) saw a benefit of the RPM system for management of their health condition. On average, patients interacted with the wearable on 61% (SD 26%) of days enrolled in the study. SHD were available on average for 78% (SD 23%) of days, and PROM data were available on 64% (SD 27%) of weeks enrolled in the study. Wearable interaction frequency, SHD, and PROM completeness were most influenced by intervention group membership.

**Conclusions:**

Our results mark a first step toward integrating RPM systems based on a consumer wearable device for primary patient input into standardized clinical workflows. They can serve as a blueprint for creating a user-friendly, reusable, interoperable, and secure RPM system that can be integrated into patients’ daily routines.

## Introduction

### Background

With constant advancements in digitalization, wearable devices such as smartwatches, fitness trackers, or chest straps are becoming an integral part of everyday life. These devices facilitate continuous recording and monitoring of patient-generated data, such as sensor-based health data (SHD) and electronic patient-reported outcome measures (PROMs). If these data were efficiently shared with physicians, they could gain a more comprehensive view of a patient’s lifestyle and longitudinal insights into a patient’s health trajectory [1]. Although most wearables are designed for the consumer market and are not primarily intended as medical devices, initial clinical trials have shown that the quality of patient-generated data from wearable consumer devices is sufficiently high to answer medical questions [2-4]. In addition, selected wearable consumer devices have been approved for various diagnostic purposes by authorities such as the US Food and Drug Administration. Especially for physical activity and cardiovascular monitoring, the development of wearables with accurate sensors is well advanced (eg, for step count as well as heart rate and electrocardiogram [ECG] measurements).

Patients show willingness to share their self-generated data with physicians, anticipating that the integration of wearables into their health care journey positively enhances their health [5]. Consequently, this holds potential for both patients and physicians to engage in collaborative health management. This can include monitoring the patient’s health status; managing risk factors; and facilitating asynchronous feedback to support effective self-management, especially in the case of chronic conditions [6]. In addition, it has the potential of reducing the frequency of needed physician consultations; improving quality of life; and, ultimately, reducing long-term treatment costs [7-10].

To exploit this potential, suitable IT systems following a patient-centric design must be available. In the literature, these systems are often referred to as *telehealth*, *telemedicine*, *eHealth*, *mHealth*, or *remote patient monitoring* (RPM) systems; the terminology is used interchangeably and inconsistently [11,12]. RPM systems must be able to process and transfer large amounts of data in an automated manner and should facilitate the integration of wearable devices into a patient’s everyday life as well as into standardized primary care and clinical research workflows. Methods for realizing such integrations are still immature due to numerous challenges in areas including patient digital literacy, data overload, interoperability, data privacy, data protection, and information security [13-16]. Overall, high usability and, consequently, high acceptance of RPM systems among patients during their treatment process is needed a priori. In addition, wearables are mostly used by healthy individuals as lifestyle devices [17] or for early disease detection in younger adults, such as in the Apple Heart Study [18] or the Fitbit Heart Study [19].

### Objectives

In its position papers on wearable-based detection of arrhythmias [20] and eCardiology [21], the German Cardiac Society and other international organizations emphasize the benefits of wearables and RPM systems for primary care and clinical research, including the treatment of heart failure. This chronic disease has a high worldwide prevalence, with an estimate of up to 64 million individuals affected. In high-income countries, it is assumed that 1% to 2% of the adult population has heart failure [22]. These patients usually have multiple contact with health care providers each year; the rehospitalization rates can approach 30% within 90 days of discharge [23]. RPM systems have been shown to detect early health deterioration of patients with chronic heart failure, triggering therapeutic interventions that could reduce rehospitalizations. Overall, approximately 30% to 40% of patients with heart failure have nonischemic cardiomyopathy, such as dilated cardiomyopathy (DCM) [24]. Patients with DCM face physical and quality of life limitations and are often young. In the past, they have been discouraged from physical activity [25]. However, exercise has, in principle, been shown to positively impact morbidity, quality of life, and patients’ psychological state, which in turn enhances their physical well-being [26-28]. Sports-related complications, such as ventricular arrhythmias, can counteract the positive effects. Therefore, the activeDCM study [29] investigated the impact of an individualized exercise program in patients with DCM. In this paper, we introduce the RPM system used in activeDCM, detailing its conceptualization, implementation, and evaluation. The objective of this study was to create a user-friendly, reusable, interoperable, and secure RPM system incorporating asynchronous feedback mechanisms using a broadly available consumer wearable device for primary patient input. In addition, this study sought to evaluate factors influencing patient acceptance of the developed RPM system.

## Methods

### Study Design

activeDCM was designed as a prospective, randomized, interventional case-control study and is described by Sedaghat-Hamedani et al [29]. The primary outcome measure of activeDCM was defined as the change in maximum oxygen uptake, whereas the secondary outcome measures focused on changes in quality of life and behavioral lifestyle. The inclusion criteria for patients were age between 18 and 75 years and diagnosis of nonischemic DCM (left ventricular ejection fraction of ≤50% and New York Heart Association Classification of I-III). The exclusion criteria were acute myocarditis or Takotsubo syndrome; known or suspected ischemic heart disease; known syndromic DCM; history of syncope, cardiac arrest, sustained ventricular tachycardia, or cardiac decompensation within the previous 3 months; contraindications for exercise testing or training; and pregnancy or breastfeeding in women. Patients were identified during initial presentations or routine examinations at the outpatient center for cardiomyopathies at Heidelberg University Hospital and were screened against the inclusion and exclusion criteria by a physician familiar with the study. Participation in the study lasted at least 12 months for each patient. On the day of enrollment, patients were randomly assigned to one of three study arms: (1) intervention group with an individualized exercise program and feedback messages (IG+), (2) intervention group with an individualized exercise program but without feedback messages (IG–), and (3) control group (CG).

### Requirements

Using the focus group methodology [30], the following requirements for a patient-centric RPM system were systematically delineated through collaborative sessions involving cardiologists (n=3), mobile health experts (n=2), and potential users (n=2) affiliated with Heidelberg University Hospital, Germany.

The functional requirements were as follows:

1. Patients should use a wearable as primary input device. They should be equipped with a wearable by the study team and not use their own device.
2. Patients should be able to record and transmit patient-generated data (ie, the SHD steps, active burned energy, heart rate, and ECG as well as the PROMs of a study-specific, weekly 7-part questionnaire and an optional exercise diary) in near real time to the study center.
3. Physicians should be able to send personalized feedback messages to patients with motivational content about the individualized exercise program.
4. Physicians should be able to review the substantial volume of transmitted patient-generated data in a streamlined format.

The nonfunctional requirements were as follows:

1. The RPM system should prioritize usability, thereby minimizing barriers to integrating the wearable into patients’ daily routines as many patients are older and a prestudy showed little penetration of wearable devices in this cohort (data not shown).
2. The RPM system should be based on medical IT standards for interoperability.
3. The RPM system should meet the regulatory requirements for data privacy and data protection as well as provide state-of-the-art information security.
4. The RPM system should be usable beyond the activeDCM study.

### Concept and Implementation

On the basis of the identified requirements, a concept of an RPM system with asynchronous feedback mechanisms was designed and subsequently implemented using an iterative approach. In each iteration, a new module of patient-generated data or for feedback messages was added to the system. Throughout the implementation process, a test environment was available, allowing for the immediate testing of each newly added module. This facilitated short feedback cycles with test participants and physicians from the activeDCM study and ensured the technical feasibility of a patient-centric RPM system.

The final concept, as well as the implementation, consisted of 4 main components: patient devices, data server, data viewer, and notification service (see the Results section). The patient devices used in this study included an iPhone (SE generation 1 or newer) and an Apple Watch (Series 4 or newer, generously provided by Apple Inc). A study-specific wearable and smartphone app was implemented using the Swift programming language (version 5.1; Apple Inc) [31] for the operating systems of both devices (ie, iOS version 14 and watchOS version 7). The extraction of the SHD (steps, active burned energy, heart rate, and ECG) was based on the Core Motion [32] and HealthKit [33] frameworks for iOS and watchOS development. The PROMs (study-specific, weekly questionnaire and optional exercise diary) were recorded by means of a self-designed user interface using Apple’s UIKit [34] framework. The transfer of data from the Apple Watch to the iPhone and vice versa was based on the iOS and watchOS Watch Connectivity protocol [35]. The data storage on the iPhone was based on the Realm database (version 5.5; MongoDB Inc) [36].

The implementation of the data server as well as the transmission of the patient-generated data was based on the standardized application programming interface (API) and the standardized data model of Health Level 7 (HL7) Fast Healthcare Interoperability Resources (FHIR) version R4 [37], as well as the Logical Observation Identifiers Names and Codes (LOINC) [38] and the Unified Medical Language System (UMLS) [39] (see the Data Model section) in the Java programming language (version 11; Oracle Corporation) [40] using the HAPI library (version 5.2) [41]. The database technology used was PostgreSQL (version 12; PostgreSQL Global Development Group) [42].

The physician data viewer component was implemented as a mobile-first TypeScript (version 4.1; Microsoft Corp) [43] web application using the vue.js framework (version 2) [44] and the chart.js library (version 2) [45]. The notification service was provided by the device manufacturer (ie, Apple Inc). Local on-device and remote push notifications for the patient devices were implemented using the User Notifications [46] framework for iOS and watchOS development.

The aforementioned version numbers correspond to the ones at the beginning of the implementation process and were regularly updated during the use of the RPM system if new applicable versions were released or APIs changed. These updates did not change any functionality or user interfaces of any component.

### Procedure

**Figure 1:**
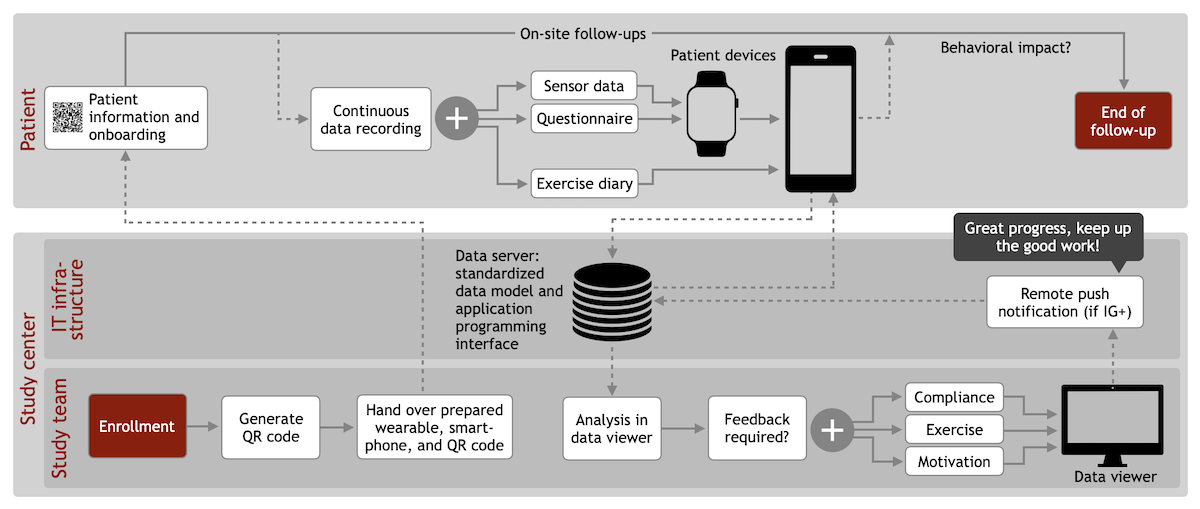
Use of the remote patient monitoring (RPM) system in the activeDCM study. After onboarding, patients are equipped with devices whose RPM system app is activated by scanning a patient-specific QR code. Patient-generated data are then continuously recorded and transmitted to the study center until the end of follow-up. Physicians analyze the data and send feedback messages to the patients’ devices if appropriate. IG+: intervention group with feedback messages.

To ensure both technical and clinical feasibility, the RPM system was used in the activeDCM study following a procedure that was developed in addition to the requirements as part of the focus group sessions. The procedure is illustrated in Figure 1. On the day of enrollment, patients were randomized to 1 of the 3 study arms and provided with a configured device bundle consisting of a consumer wearable (Apple Watch Series 4 or newer) and a smartphone (iPhone SE generation 1 or newer) on which the corresponding app of the RPM system was preinstalled. Patients were not provided with a mobile data volume contract for the devices; instead, they were required to set up a Wi-Fi connection at their home. After a thorough patient educational session regarding the study and the RPM system covering instructions on use of the wearable and smartphone, including the RPM-specific app and how to launch a workout session with resultant increased frequency of activity measures, patients signed the written informed consent form. After that, they were onboarded to the app by scanning a patient-specific QR code using the smartphone’s camera. From this point forward, the required SHD were continuously recorded by the wearable. The synchronization of the recorded data with the study center was confirmed by patients through a short daily interaction with the wearable. In addition, patients were asked to answer 1 randomly selected question daily from the study-specific, weekly 7-part PROM questionnaire [47], requiring a second daily interaction. To remind patients of these 2 mandatory interactions with the wearable, they were alerted daily at 11 AM and 5 PM with local on-device push notifications sent directly by the wearable app. An interaction with the smartphone was not mandatory but could take place to keep an optional PROM exercise diary, which was synchronized with the study center as well. Each patient was part of the activeDCM study over a period of at least 12 months. Patients were allowed to withdraw at any time from the study. During participation in the study, patient-generated data were collected and transmitted from the wearable via the smartphone to the study center, which in turn provided feedback messages to the patients in the corresponding intervention group (IG+). Feedback messages containing motivational content about the individualized exercise program were sent by a physician using the study center’s data viewer approximately every 7 to 10 days using remote push notifications.

### Evaluation

The evaluation of the RPM system was conducted in 2 parts. First, to assess patients’ subjective perspectives on the RPM system, a 2-part evaluation questionnaire was used to address the following two end points: (1) the first part of the evaluation questionnaire asked about experience (no: without previous device experience [Exp–], yes: with previous device experience [Exp+]) using Apple devices (iPhone or Apple Watch) before participating in the activeDCM study and used a German version [48] of the standardized System Usability Scale (SUS) [49] to assess user-friendliness, and (2) the second part contained 5 self-designed questions to assess patients’ attitudes toward the use of an RPM system for disease management.

Both parts of the evaluation questionnaire—the SUS and self-designed questions—used a 5-point ordinal Likert scale with each item ranging from *strongly disagree* to *strongly agree*. An SUS value of >68 was considered user-friendly [50]. The evaluation questionnaire was administered to each patient at completion of the activeDCM study protocol after at least 12 months and can be found in Multimedia Appendix 1.

Second, to assess patients’ objective use of the wearable and the interaction with the study-specific wearable and smartphone app, three additional end points were evaluated: (3) to assess *wearable interaction frequency*, the number of days with at least one wearable interaction (either SHD transmission or answering a PROM questionnaire item) was compared with the total number of days that a patient was enrolled in the study; (4) *SHD completeness* was assessed by comparing the days with available SHD on the data server with the total number of days that a patient was enrolled in the study; and (5) finally, to assess *PROM completeness*, the number of completed and available PROM questionnaires on the data server was compared to the total number of PROM questionnaires expected to be completed during the study period (1 per week).

The statistical analysis of all 5 end points was conducted using descriptive statistics incorporating box plots. Continuous variables were described using mean and SD, and categorial variables were described using absolute and relative frequencies. Assessment of differences in the patient demographics of the evaluation groups was performed using the Kruskal-Wallis test [51] for continuous variables. For categorical variables, the Pearson chi-square test [52] was used.

As the number of days for which each patient participated in the activeDCM study varied, we calculated relative values for end points 3, 4, and 5 for each patient. We then described these relative values using mean and SD and used them for further statistical analysis. For comprehensiveness, we reported the ratio of mean absolute values in days and weeks across all patients for end points 3, 4, and 5 as well. However, the mean of the relative values offers a more detailed understanding of the differences between patients and produces more accurate results than the ratio of mean absolute values. It is important to note that these 2 measures are not mathematically equivalent.

Furthermore, bias-corrected and accelerated bootstrapped multiple linear regression [53] was performed to investigate evaluation group differences in the SUS, patient wearable interaction frequency, and SHD or PROM completeness. Thus, the outcome (dependent) variables were the SUS score, patient wearable interaction frequency, and SHD or PROM completeness after activeDCM study protocol completion. As independent variables, age in years at activeDCM study protocol completion, sex, device experience (no: Exp–, yes: Exp+), and study arm membership (CG, IG–, IG+) were chosen before the analysis. The analysis involved 10,000 bootstrap resampling distributions applying a CI of 95%. CIs not including 0 were considered as significant (*P*<.05). The analysis was conducted using Python (version 3.10; Python Software Foundation) [54] using the packages *scipy.stats.kruskal* (version 1.11.1) [55], *scipy.stats.chi2_contingency* (version 1.11.1) [56], *scipy.stats.bootstrap* (version 1.11.1) [57], and *statsmodels.regression.linear_model.OLS* (version 0.14.0) [58].

Due to the exploratory characteristics of this study, the analysis is intended for hypothesis generation only; thus, *P* values were not adjusted for multiplicity, and *P*<.05 was regarded as significant.

### Ethical Considerations

The activeDCM study, as well as its RPM system concept, its implementation, and its evaluation method, received ethics approval from Heidelberg University’s research ethics committee (reference numbers S-740/2018 and S-740/2021). The study is registered on ClinicalTrials.gov (NCT04359238). It adheres to the principles of the Declaration of Helsinki and good clinical practice guidelines. Participation in the activeDCM study was voluntary and patients received no compensation. An Apple Watch (Series 4 or newer) and an iPhone (SE generation 1 or newer) were provided during participation in the activeDCM study. After study completion these devices had to be returned to the study team. A written informed consent form was signed by each patient participating in the study. In addition, the RPM system’s data protection concept and its security mechanisms were audited by the data protection officer of Heidelberg University Hospital. No deficiencies were identified regarding data privacy, data protection, and information security.

## Results

### Concept

**Figure 2:**
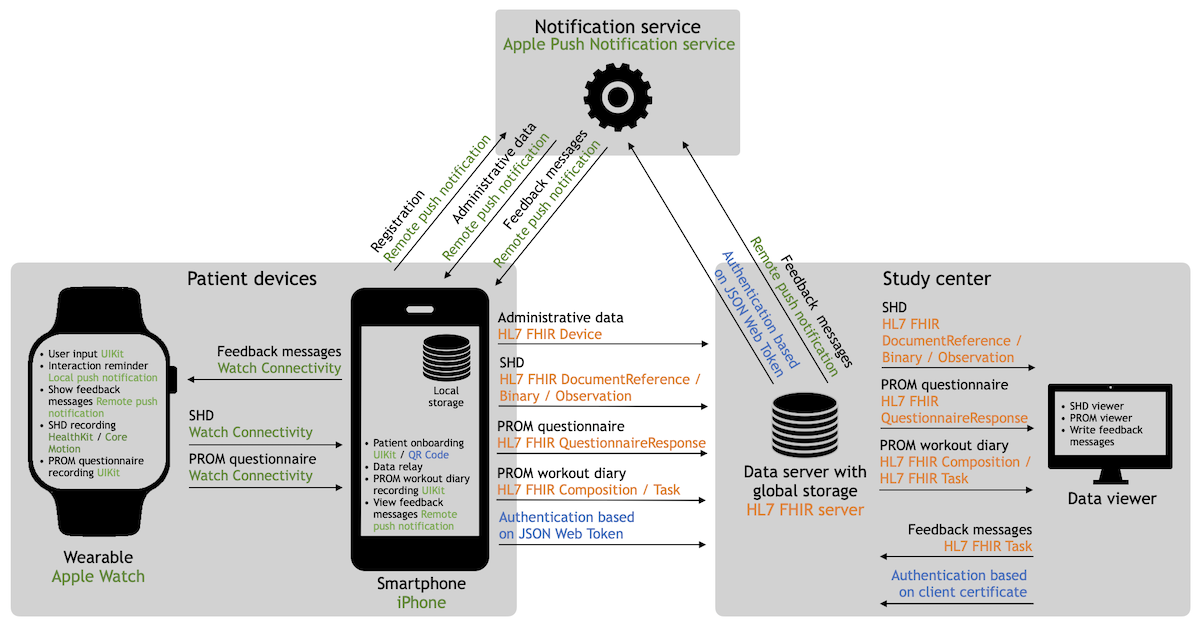
Architecture of the remote patient monitoring system with asynchronous feedback mechanisms describing the 4 main components and their interaction. Black writing shows the initial concept, and colored writing shows concepts realized subsequently as part of the implementation. The services, frameworks, and protocols of the Apple ecosystem are shown in green; the standardized data model based on Health Level Seven (HL7) Fast Healthcare Interoperability Resources (FHIR) are shown in orange; and the authentication mechanisms are shown in blue. PROM: patient-reported outcome measure; SHD: sensor-based health data.

On the basis of the identified requirements, a concept of a patient-centric RPM system with asynchronous feedback mechanisms was designed. It consisted of 4 interoperable components: patient devices (ie, a smartphone and a connected consumer wearable device, both having a corresponding RPM system app installed), a data server, a data viewer, and a notification service. A graphical representation of the concept and its implementation can be found in Figure 2 summarizing the key features and functionalities of the individual components. In this concept, the smartphone was used for patient onboarding and as a data relay with local storage for data transmission to the data server, which was located at the study center. The wearable was intended to be the patient’s primary input device. It was used to record SHD as well as collect PROMs to transmit these patient-generated data to the data server, relayed via the smartphone. In addition, the wearable was supposed to remind patients about necessary interactions and display personalized feedback messages, which were received from the notification service and forwarded by the smartphone. The data server was responsible for global storage of patient-generated data and feedback messages in a standard-compliant, interoperable manner. In addition, it made patient-generated data available to the data viewer. The data viewer, operated at the study center, allowed physicians to analyze patient-generated data and send personalized feedback messages to the data server. These feedback messages were stored on the data server and transmitted to the notification service. The notification service was responsible for identifying the smartphone that would receive the forwarded feedback messages. For the notification service to uniquely identify a smartphone, the latter must undergo an initial registration process during patient onboarding. At the end of this process, administrative data were generated and forwarded from the smartphone to the data server. The data server used the administrative data to identify the smartphone when forwarding feedback messages to the notification service.

### Implementation

#### Overview

**Figure 3:**
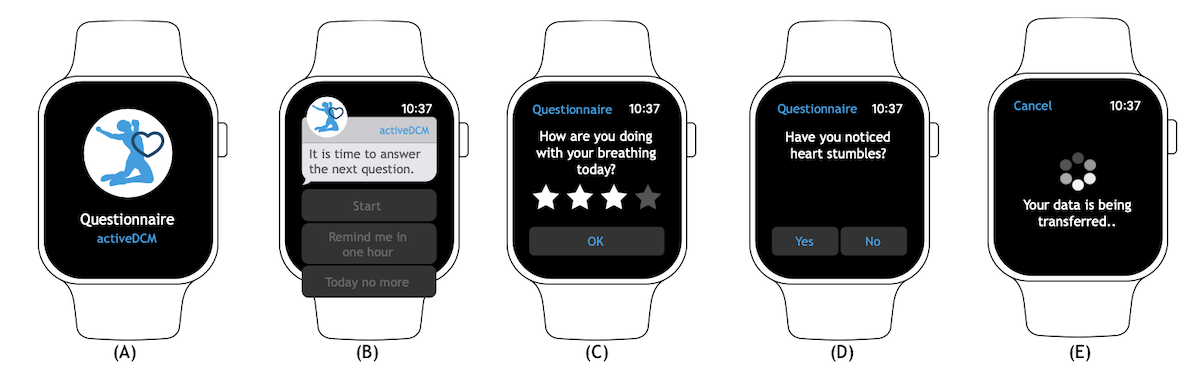
Screenshots of the Apple Watch app. (A) and (B) show the daily notification to remind patients about answering 1 question of the 7-part weekly patient-reported outcome measure (PROM) questionnaire. (C) and (D) show the user interface to enter 1 of 2 different answer types for the PROM questionnaire—(C) allows for entering a star rating between 1 and 4, and (D) allows for entering a Boolean answer using yes or no. (E) shows the user interface during the transmission of the patient-generated data to the study center.

The described concept was subsequently implemented for the activeDCM study. For patient devices, an iPhone (SE generation 1 or newer) was used as the smartphone, and an Apple Watch (Series 4 or newer) was used as the wearable for which a study-specific app was implemented. Selected screenshots of the Apple Watch app can be found in Figure 3. The onboarding of a patient to the RPM system was based on scanning a patient-specific QR code on the iPhone’s app. After that, SHD recording started immediately on the Apple Watch. Security mechanisms permitting data access and extraction from HealthKit exclusively if the device was unlocked forced the extraction of SHD on the Apple Watch app, which must be unlocked during interaction. Each type of SHD and PROM was implemented in its own module so that each module could be activated and deactivated independently of one another. To remind patients to answer the daily question of the PROM questionnaire and transfer patient-generated data to the study center, local on-device notifications were implemented directly as part of the Apple Watch app.

Transmission of patient-generated data from the Apple Watch to the iPhone was executed using background processes triggered by interactions with the Apple Watch on-device notifications. Transmission errors between Apple Watch and iPhone were displayed to the patient in the user interface, cached, and retried on the next transmission. Once the patient-generated data had been transmitted to the iPhone, they were forwarded again through background processes to the data server without any user interaction. This meant that no patient interaction with the iPhone was necessary. It could remain plugged into a power supply socket for the duration of the study and did not have to be carried around. Data transmissions used the standardized HL7 FHIR API provided by the data server. Each data transmission from the iPhone to the data server was recorded with an audit message, which could be viewed by a patient on the user interface of the iPhone app. If a transmission error occurred, the patient-generated data were temporarily stored in an encrypted local database, and the transmission was retried during the next scheduled data transfer to the data server.

The data server provided global storage of patient-generated data and feedback messages using standardized HL7 FHIR R4 resources as a data model. In addition, it executed algorithms periodically to check the date and time of the last data transmission of each patient. If >5 days had passed since a patient’s last data transmission, an automated feedback message was sent to the patient’s devices with a request to synchronize data.

The data viewer retrieved patient-generated data via the HL7 FHIR API and displayed them through a self-designed user interface. Data transmitted by a patient could be viewed at several levels of granularity (ie, data for a single day, data for 2 weeks, data for 1 month, or all recorded and transmitted data since study enrollment). Depending on the data type, they were presented using different visualization formats: (1) bar plots illustrated various granularities of active burned energy and step data, (2) scatter plots were used for daily heart rate views, and (3) box plots were used for 2 weekly and all-data heart rate views.

**Figure 4:**
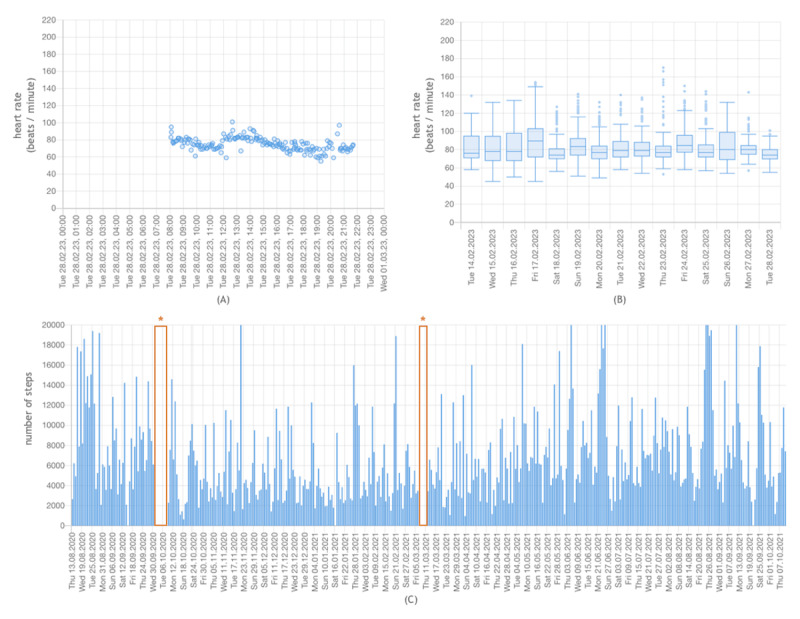
Screenshots of the data viewer application for 1 patient; (A) shows the recorded and transmitted heart rate data of a single day as a scatter plot; (B) shows the recorded and transmitted heart rate data over 2 weeks as daily box plots; (C) shows the recorded and transmitted step counts for all days included in the study as a zoomable bar plot, where asterisks indicate days without any recordings or transmissions, for example, because the wearable was not worn.

If all the data of a patient since study enrollment were displayed, the visualization was zoomable so that a closer look at areas of interest was possible. Selected screenshots of the data viewer user interface can be found in Figure 4. A screencast showing the functionality of the data viewer (with a German user interface) can be found in Multimedia Appendix 2.

To receive remote push notifications from the study center, the patient’s iPhone registered itself with Apple Push Notification service (APNs) directly after onboarding. APNs provided a device-specific identifier, which was forwarded by the iPhone app to the data server of the study center as administrative data. On the basis of the device identifier, the data server could forward a feedback message received from the data viewer to the APNs server after the message was transformed from the standardized HL7 FHIR format to the APNs-required proprietary format. The device identifier then enabled the APNs server to forward the feedback message as a remote push notification to the patient’s devices. Each feedback message was recorded, in addition to an audit message on the iPhone, and could be viewed by a patient in the app’s user interface.

#### Data Model

The data model was based on standardized HL7 FHIR R4 resources to establish syntactic interoperability. A patient was identified by a *Patient* resource containing the patient’s study pseudonym. The *Device* resource stored the device-specific identifier provided by APNs, which was needed to send and retrieve feedback messages via remote push notifications and linked to the *Patient* resource. Feedback messages were modeled using the *Task* resource, which contained information such as title, message, category, and recipient, which was a reference to the *Patient* resource. The SHD steps, active burned energy, and heart rate were modeled using the *Observation* resource. For steps and active burned energy, the recorded data were summarized in 1-hour intervals. For heart rate, 25 consecutive measurements were listed and transferred in 1 *Observation* resource to reduce network calls. The *DocumentReference* resource was used to store metadata related to ECG data, such as recording date, classification, and average heart rate. In addition, it referenced a *Binary* resource to transport the actual ECG recording (512 Hz; 30 seconds) as millivolt values in a CSV file. The PROM questionnaire was based on the *Questionnaire* resource, which contained the questions to be answered weekly, and the *QuestionnaireResponse* resource, which stored a patient’s completed questionnaire. The exercise diary was based on the *Composition* resource, which stored the date of a completed workout and was linked to several *Task* resources containing information about the executed strength and endurance exercises during the workout.

To establish semantic interoperability with other clinical information systems (eg, from primary care), the SHD were annotated using LOINC codes, and the PROM questionnaire was annotated using postcoordinated UMLS codes. For semantic annotation of the PROM exercise diary, we used a self-defined code system based on the exercise program. HL7 FHIR profiles, concrete resource examples, and validation information for the data model can be found in Multimedia Appendix 3.

#### Security, Authentication, and Authorization

For general security, all messages exchanged between the components of the RPM system were encrypted using Transport Layer Security [59] using a minimum version of 1.2. The Realm database used to cache patient-generated data on the iPhone was encrypted using the Advanced Encryption Standard-256+Secure Hash Algorithm 2 algorithm and could not be analyzed if the device was lost. The encryption key of the database was stored in the secure Keychain [60] of the iPhone and could only be accessed through the iPhone app.

Authentication and authorization to the RPM system distinguished between the roles of patient and study center staff using distinct technical approaches for each. For the patient role, the QR code scanned during onboarding contained a signed JSON Web Token (JWT) [61] valid for study enrollment, storing authentication information and the patient’s pseudonym in the iPhone’s secure Keychain. This JWT was used when transmitting patient-generated data, ensuring authentication and data provenance. The role of study center staff, authenticated using X.509 client certificates [62], could retrieve patient-generated data and stored push notifications on the data server. Revoking compromised certificates or JWTs was managed via configuration options on the data server. The server’s authentication to APNs used a JWT as well that was signed and encrypted by the study center data server using a private key from Apple that was revocable on the Apple developer website if compromised.

### Evaluation

The evaluation of the RPM system was carried out with patients who completed the activeDCM study protocol between October 2021 and February 2024 (N=110). A total of 13.6% (15/110) of the patients were excluded from the evaluation because of missing answers in the evaluation questionnaire on experience or SUS answers. The evaluated cohort consisted of 95 patients (n=28, 29% female) with a mean age of 50 (SD 12) years at the end of study participation. Of these 95 patients, 39 (41%) had previous experience using Apple devices (Exp+, n=30, 77% using iPhone only and n=9, 23% using iPhone and Apple Watch), and 56 (59%) had no experience (Exp–) at the time of enrollment in the study. A total of 26% (25/95) of the patients were randomized into the intervention group with an individualized exercise program and feedback messages (IG+), 36% (34/95) were randomized into the intervention group with an individualized exercise program but without feedback messages (IG–), and 38% (36/95) were randomized into the CG study arm. The mean enrollment time of these patients in the activeDCM study was 396 (SD 39) days, which corresponds to 56 (SD 5) weeks. The baseline characteristics of the evaluated patient cohort are summarized in Table 1.

**Table 1:** Baseline characteristics of patients by device experience and study arm membership in the activeDCM study (N=95).

Variable		Total sample	Exp–^a^ (n=56)	Exp+^b^ (n=39)	CG^c^ (n=36)	IG–^d^ (n=34)	IG+^e^ (n=25)
**Sex, n (%)^f^**							
	Male	67 (71)	40 (71)	27 (69)	27 (75)	24 (71)	16 (64)
	Female	28 (29)	16 (29)	12 (31)	9 (25)	10 (29)	9 (36)
Age (y)^g^, mean (SD; range)		50 (12; 23-66)	51 (10; 23-66)	49 (13; 24-66)	50 (11; 24-65)	48 (13; 23-66)	53 (11; 24-66)
Time in study (d)^h^, mean (SD; range)		396 (39; 315-625)	398 (44; 315-625)	394 (30; 330-456)	402 (50; 315-625)	395 (28; 336-448)	389 (31; 330-484)

^a^Exp–: without previous device experience.

^b^Exp+: with previous device experience.

^c^CG: control group.

^d^IG–: intervention group without feedback messages.

^e^IG+: intervention group with feedback messages.

^f^Device experience: Pearson *χ*^2^_1_*P*>.99; study arm: Pearson *χ*^2^_2_*P*=.65.

^g^Device experience: Kruskal-Wallis test *P*=.58; study arm: Kruskal-Wallis test *P*=.29.

^h^Device experience: Kruskal-Wallis test *P*=.95; study arm: Kruskal-Wallis test *P*=.53.

**Figure 5:**
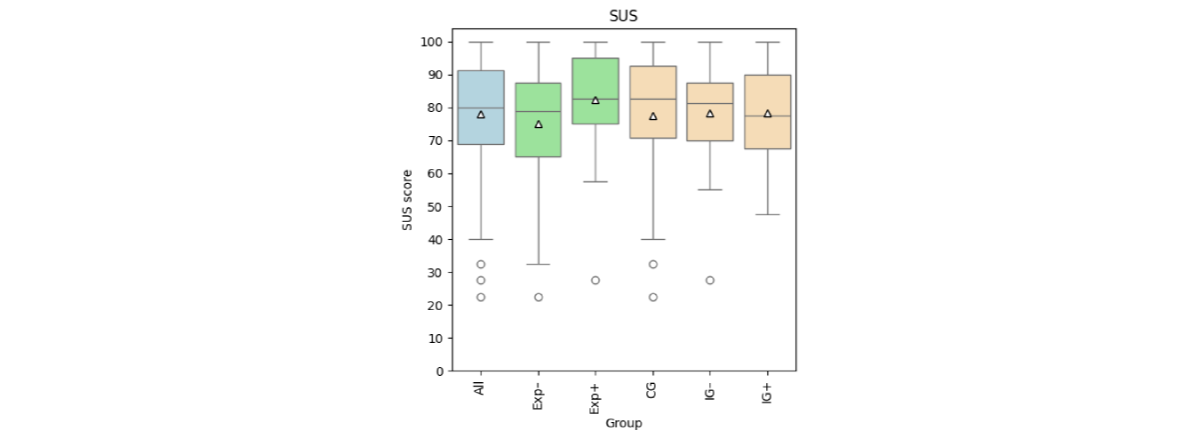
Results of the wearable and smartphone app usability analysis using the System Usability Scale (SUS) displayed as box plots. All data combined are shown in blue, experience using Apple devices is shown in green, and study arm membership is shown in orange. Triangles show mean values. CG: control group; Exp–: without previous device experience; Exp+: with previous device experience; IG–: intervention group without feedback messages; IG+: intervention group with feedback messages.

The results of the patients’ subjective perspective on the RPM system based on the evaluation questionnaire were divided into 2 end points. The results of the first end point, analyzing the usability of the wearable and smartphone app based on the SUS, are summarized using box plots in Figure 5. The app achieved a mean SUS score of 78 (SD 17). A total of 25% (24/95) of the patients reported an SUS score of <68 (insufficient usability), of whom 75% (18/24) were aged >50 years and 75% (18/24) had no previous experience using Apple devices (Exp–). Patients with previous experience using Apple (Exp+) devices rated usability more highly, with a mean SUS score of 82 (SD 15), compared with patients without any experience (Exp–), who reported a mean SUS score of 75 (SD 18). There were no major differences among patients in the study arms. Patients in the intervention group with feedback messages (IG+) reported a mean SUS score of 78 (SD 15), patients in the intervention group without feedback messages (IG–) reported a mean score of 78 (SD 15), and patients in the CG reported a mean score of 77 (SD 20).

As shown in Table 2, the bias-corrected and accelerated bootstrapped multiple linear regression analysis of the SUS scores resulted in a significant effect for those with previous experience (Exp+) with a 95% CI of 0.25-13.47 and an estimate of 6.75. This means that patients with Apple device experience were estimated to rate the usability of the wearable and smartphone app 6.75 points higher on the SUS than patients without Apple device experience. This is similar to the measured differences in SUS scores between those without and with previous experience (7-point difference). Age approached significance with a 95% CI of −0.46 to 0.14. The estimate of −0.19 showed that a 40-year age difference between 2 patients was estimated to result in a 7.60-point worse SUS score for the wearable and smartphone app from the older patient.

**Table 2:** Bias-corrected and accelerated bootstrapped multiple linear regression analysis of the System Usability Scale (SUS) scores, the wearable interaction frequency (percentage of days enrolled in the study), the sensor-based health data (SHD; percentage of days enrolled in the study), and patient-reported outcome measure (PROM) completeness (percentage of weeks enrolled in the study).

Variable				Estimate, mean (95% CI)
**SUS score**				
	Sex (male)			−3.61 (−10.35 to 4.54)
	Age			−0.19 (−0.46 to 0.14)
	Exp+^a^			*6.75* *(0.25 to 13.47)* ^b^
	**Group**			
		IG+^c^	0.95 (−7.80 to 10.10)	
		IG–^d^	−0.37 (−7.81 to 8.00)	
**Wearable interaction frequency (%)**				
	Sex (male)			−7.06 (−17.7 to 3.93)
	Age			0.39 (−0.08 to 0.84)
	Exp+			−1.38 (−10.81 to 8.65)
	**Group**			
		IG+	6.08 (−6.54 to 19.51)	
		IG–	*13.53 (1.02 to 25.4)* ^b^	
**SHD completeness (%)**				
	Sex (male)			−8.22 (−16.39 to 0.32)
	Age			*0.45 (0.06 to 0.95)* ^b^
	Exp+			5.36 (−2.44 to 13.88)
	**Group**			
		IG+	*12.18 (1.65 to 22.43)* ^b^	
		IG–	*11.73 (1.17 to 21.86)* ^b^	
**PROM completeness (%)**				
	Sex (male)			−5.49 (−17.14 to 5.71)
	Age			0.42 (−0.04 to 0.92)
	Exp+			−1.43 (−12.03 to 9.31)
	**Group**			
		IG+	7.17 (−7.39 to 20.9)	
		IG–	12.22 (−0.37 to 24.29)	

^a^Exp+: with previous device experience.

^b^CIs not including 0 were considered as significant (*P*<.05).

^c^IG+: intervention group with feedback messages.

^d^IG–: intervention group without feedback messages.

**Figure 6:**
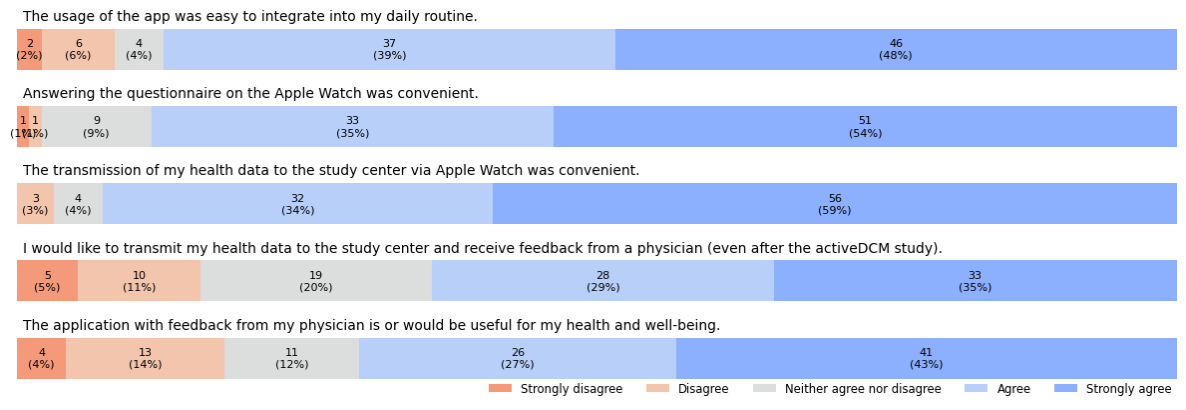
Results of the 5 self-designed questions regarding the patients’ attitudes toward using the remote patient monitoring system as well as their participation in the activeDCM study. Responses to the statements were provided by patients using a 5-point ordinal Likert scale ranging from strongly disagree in orange to strongly agree in blue.

The results of the second evaluation questionnaire end point, regarding the 5 self-designed questions about the patients’ attitudes toward using the RPM system as well as their participation in the study, are shown in Figure 6. A total of 87% (83/95) of the patients (strongly) agreed that the use of the app was easy to integrate into their daily routine. Similarly, 88% (84/95) and 93% (88/95) of the patients considered answering the PROM questionnaire and transmitting their SHD to the study center via Apple Watch as convenient, respectively. In total, 64% (61/95) of the patients would like to submit their health data to the study center and receive feedback from a physician (even after the activeDCM study), and the app with feedback from their physician was or would be useful for 71% (67/95) of the patients regarding their health and well-being.

**Figure 7:**
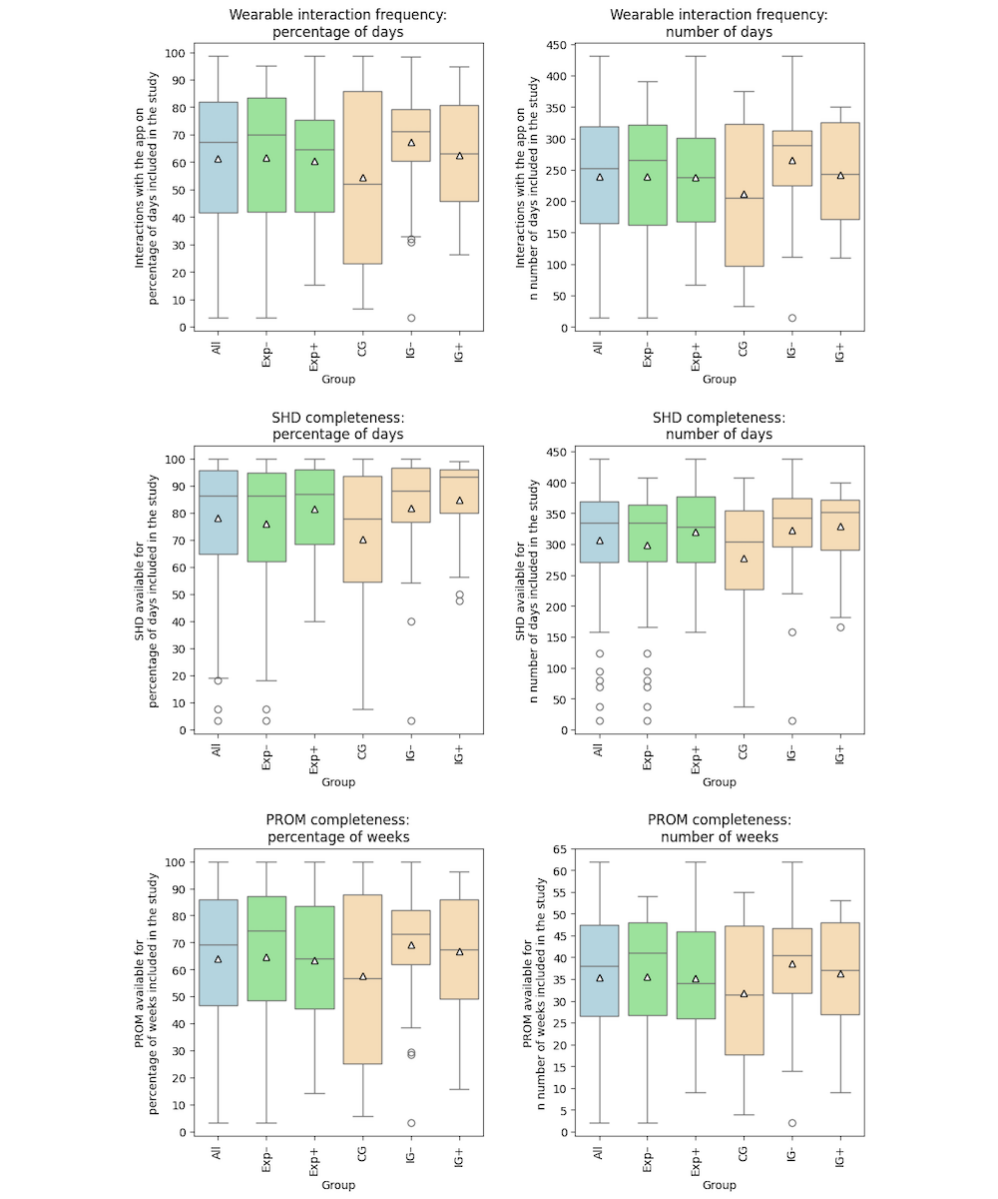
Results of patients’ wearable interaction frequency as well as completeness of recorded and transmitted patient-generated data classified into sensor-based health data (SHD) and electronic patient-reported outcome measures (PROMs) of the wearable and smartphone app as box plots. All data combined are shown in blue, experience using Apple devices is shown in green, and study arm membership is shown in orange. Triangles show mean values. CG: control group; Exp–: without previous device experience; Exp+: with previous device experience; IG–: intervention group without feedback messages; IG+: intervention group with feedback messages.

In Figure 7, the results of patients’ objective use of the wearable device are summarized using box plots, showing wearable interaction frequency and SHD as well as PROM completeness for end points 3, 4, and 5. On average, patients interacted with the wearable app on 61% (SD 26%) of the days that they were enrolled in the study, which corresponds to 239 (SD 99) of 396 (SD 39) days. On average, SHD were available for 78% (SD 23%) of the days that patients were enrolled in the study, which corresponds to 307 (SD 87) of 396 (SD 39) days. In terms of PROM questionnaires answered, data were available on average for 64% (SD 27%) of the weeks that the patients were enrolled in the study, which corresponds to 35 (SD 15) of 56 (SD 5) weeks. There were no major differences between those without and with previous experience (wearable interaction frequency: mean 62%, SD 27% of days [240, SD 103 of 398, SD 44 days] vs mean 60%, SD 23% of days [238, SD 91 of 394, SD 30 days], respectively; SHD completeness: mean 76%, SD 25% of days [299, SD 97 of 398, SD 44 days] vs mean 81%, SD 17% of days [319, SD 69 of 394, SD 30 days], respectively; PROM completeness: mean 65%, SD 28% of weeks [36, SD 15 of 56, SD 6 weeks] vs mean 63%, SD 24% of weeks [35, SD 14 of 56, SD 4 weeks], respectively). Only the dispersion of the data was slightly narrower for those with previous experience (Exp+). Regarding the 3 different study arms, the interaction with the wearable was more frequent and patient-generated data completeness was higher in both the IG– (wearable interaction frequency: mean 67%, SD 20% of days [266, SD 84 of 395, SD 28 days]; SHD completeness: mean 82%, SD 20% of days [322, SD 80 of 395, SD 28 days]; PROM completeness: mean 69%, SD 21% of weeks [39, SD 12 of 56, SD 4 weeks]) and IG+ (wearable interaction frequency: mean 62%, SD 20% of days [242, SD 78 of 389, SD 31 days]; SHD completeness: mean 85%, SD 16% of days [330, SD 63 of 389, SD 31 days]; PROM completeness: mean 67%, SD 23% of weeks [36, SD 12 of 55, SD 4 weeks]) than in the CG (wearable interaction frequency: mean 54%, SD 31% of days [212, SD 115 of 402, SD 50 days]; SHD completeness: mean 70%, SD 26% of days [277, SD 99 of 402, SD 50 days]; PROM completeness: mean 58%, SD 32% of weeks [32, SD 17 of 57, SD 7 weeks]). In both the IG+ and IG–, the dispersion of the data was smaller than in the CG.

The data volume recorded and transferred per patient to the data server varied based on health status, physical activity levels, and workout duration, which influenced both recorded data point density and the number of ECG recordings. On average, the data volume amounted to approximately 300 MB per patient and year in the HL7 FHIR database on the data server.

The bias-corrected and accelerated bootstrapped multiple linear regression analysis (Table 2) resulted in a significant influence of IG– membership on the wearable interaction frequency and SHD completeness end points and approached significance for the PROM completeness end point (wearable interaction frequency estimate: 13.53%, 95% CI 1.02%-25.4%; SHD completeness estimate: 11.73%, 95% CI 1.17%-21.86%; PROM completeness estimate: 12.22%, 95% CI –0.37% to 24.29%). This means that, on average, a patient in the IG– was expected to interact with the wearable app on 13.35% more days enrolled in study than a patient in the CG and that both SHD and PROM data were expected to be available on 11.73% more days and 12.22% more weeks that a patient was enrolled in the study, respectively.

## Discussion

### Principal Findings

In this paper, we presented a concept, implementation, and evaluation of a reusable RPM system following a patient-centric approach for RPM without direct physician-patient contact using asynchronous feedback mechanisms. The system was based on patient-generated data such as SHD and PROMs. It leveraged a consumer wearable device (smartwatch) instead of a smartphone as the primary input device. The implementation of the concept and its subsequent use as part of the activeDCM randomized controlled clinical trial demonstrated that the identified challenges and requirements of a sophisticated precision digital health trial could be addressed. The implementation relied on wearable devices and smartphones from Apple Inc and their notification service, but the concept should be versatile enough to be used with devices based on other operating systems, such as the Android-based Wear OS for the Samsung Galaxy Watch or the Google Pixel Watch.

Decisions during RPM system conceptualization and implementation aimed to minimize barriers to use, enhancing usability and ensuring high patient acceptance as well as compliance. The study team established a device preparation and distribution scheme providing patients with ready-to-use devices, thereby reducing the configuration efforts to scanning an onboarding QR code. Patient education sessions focused not only on the medical aspects of the study but also on digital literacy and provided written and video-based material about the wearable and smartphone app, requiring additional effort from patient educational sessions. Opting for a wearable as the primary input device likely played a pivotal role in seamlessly integrating the RPM system into the patients’ daily routines. Its proximity to the patient and its intuitive interaction, aligned with the chosen device, operating system, and study-specific app, contributed to a successful integration and reduced the interaction time to a few seconds per day. To enhance the wearable and smartphone app usability, we implemented only the essential functionalities in a straightforward, simplistic manner, trying to reduce the interactions for these functionalities to a minimum. This was significantly shaped by the official human interface guidelines provided by Apple [63,64]. Storing authentication information during onboarding further streamlined data transmissions, eliminating the need for patients to remember usernames and passwords.

The Apple Watch sensors capture heart rate measurements in an interval of seconds during an activity session [65], and active burned energy and step data can be extracted in hourly intervals. Given this vast amount of data points, the physician-centered data viewer prioritized simplicity, presenting summarized information. In total, 3 visualization granularity levels allowed physicians to analyze patient-generated data, identifying short-, medium-, and long-term trends, and compare data from multiple patients effortlessly. Direct remote push notifications from the data viewer interface streamlined physician support, eliminating the need to switch between various communication channels, as observed in other wearable-based studies [66,67].

The HL7 FHIR standard enhanced medical data exchange by addressing health care challenges beyond the capabilities of previous standards, such as HL7 version 2, HL7 version 3, and Clinical Document Architecture [68]. Therefore, HL7 FHIR’s standardized API and data models used in combination with the LOINC and UMLS medical terminologies ensured syntactic and semantic interoperability with other clinical information systems. However, complete integration with other clinical information systems requires these systems to provide standardized interfaces as well. Without these interfaces, integration becomes more complex, necessitating adaptations and mappings. In Germany, legislative efforts are promoting the adoption of HL7 FHIR interfaces for clinical information systems [69].

The wearable and smartphone app, data server, and data viewer implementation were not bound to a specific medical domain and could be used independently beyond the activeDCM study as long as the counterpart supported the HL7 FHIR standard as well. The components of the wearable and smartphone app and the data viewer were built iteratively in a modular system and, thus, can be adapted for further studies and other diseases such as diabetes or chronic obstructive pulmonary disease without major effort. If required, new modules could be implemented, integrating additional sensors and data types. Similarly, not required data types could be removed easily.

By equipping patients with dedicated devices, it was possible to ensure that data transmissions only took place in a pseudonymized form. During the educational session, patients were briefed on the RPM system’s privacy and security mechanisms, emphasizing the storage of only the pseudonym on devices after handover. This ensured that no data were stored in a cloud solution provided by the manufacturer of the devices. As such, the smartphone and wearable were setup using Apple Configurator 2, and the app was deployed using the over-the-air method without the need to sign in using an Apple ID. Furthermore, by separating patient identification from device identification, feedback messages could be sent via the manufacturer’s notification service without having to know the patient’s identity or pseudonym. The connection of the pseudonym to the device could only be established on the data server.

The SUS evaluation, resulting in a mean score of 78 (SD 17), indicated that the wearable and smartphone app was considered user-friendly by patients [50]. The significant difference between patients with and without experience using Apple devices (estimate: 6.75, 95% CI 0.25-13.47) suggests that patients are generally more comfortable using devices they are familiar with. However, to allow use of wearables from different manufacturers in the RPM system, achieving comparable sensor accuracies for the same SHD recordings would be essential, but the literature shows that there are measurable differences [70,71], posing challenges for physicians in comparing patient data and providing feedback. Despite the additional effort to explain the devices and study-specific app, patient age had an influence on the SUS score approaching significance (estimate: −0.19, 95% CI –0.46 to 0.14). Insufficient usability (SUS score of <68; 24/95, 25% of the patients) was mainly reported by patients aged >50 years (18/24, 75%) and with no previous Apple device experience (18/24, 75%). This suggests that the challenges faced by these individuals may not have been due to problems with the usability of the wearable and smartphone app but rather with the general use of the devices. These findings are consistent with results reported by Sonderegger et al [72] and illustrate the importance of digital literacy and the supplementary material provided for patients during the onboarding process.

On average, patients engaged with the wearable more than every other day (61%, SD 26% of days, 239, SD 99 of 396, SD 39 days). For SHD recording and transmission, as few as 1 interaction per week would suffice for complying with the activeDCM study protocol. Complete datasets would still be transferred as Apple Watch sensors store SHD for up to 10 days. The data server algorithm that monitored transmission frequency and sent automatic reminders likely played a crucial role in obtaining complete datasets. The measured user-friendliness was also reflected in the completeness of SHD data, which was available on average for 3 out of 4 days (78%, SD 23% of days, 307, SD 87 of 396, SD 39 days). This aligns with the results by Werhahn et al [73], where heart rate measurements and step counts based on a worn Apple Watch were recorded on 84.8% and 83.5%, respectively, of days that patients were included in their study. However, the monitoring period lasted only 2 months, which is significantly shorter than that in activeDCM. The average completeness of PROM questionnaires showed similar results, which were sufficient to see relevant health changes in patients and corresponds to a transmission of a complete PROM questionnaires more than every second week (mean 64%, SD 27% of weeks, 35, SD 15 of 56, SD 5 weeks).

Gaps in data, whether SHD or PROMs, could be attributed to various factors, such as illness, hospitalization, vacation, or lapses in wearing the Apple Watch. Technical issues, poor internet connection, or oversight in configuring the patient device’s Wi-Fi connection at home may also have resulted in missing patient-generated data.

However, home-based Wi-Fi connection did not restrict patient recruitment, the daily use of the wearable and smartphone app, or recording of patient-generated data. Data were still collected even without a current Wi-Fi connection. The Wi-Fi connection was only necessary for data transmission to the study center, thereby transmitting all recorded data since the last transmission. A mobile data volume contract is possible for data transfer, enabling transfers outside the patient’s home. However, as the devices and the wearable and smartphone app can temporarily store patient-generated data until they can be transferred to the data server, data completeness would remain unchanged.

There was a notable difference in the evaluation results of objective RPM use between the CG, IG–, and IG+, showing significance for wearable interaction frequency (estimate: 13.53%, 95% CI 1.02%-25.4%) and SHD completeness (estimate: 11.73%, 95% CI 1.17%-21.86%), as well as approaching significance for PROM completeness (estimate: 12.22%, 95% CI −0.37%-24.29%) in the IG– in comparison with the CG. This suggests the presence of a Hawthorne effect [74] for increased RPM system use as anticipated given the expected behavioral impact of the activeDCM study in the intervention groups. Within each randomized controlled trial, a potential bias exists in the case of a Hawthorne effect that could have increased compliance with the treatment and the use of the wearables in this study. To study the use of the RPM system in real-world settings, another design of an observational trial would be needed.

The inferior performance of the IG+ (only significant SHD completeness with an estimate of 12.18%, 95% CI 1.65%-22.43% compared with the CG) in comparison with the IG– may be attributed to more patients with no Apple device experience and more patients aged >50 years in the IG+ compared with the IG–.

Positive responses from patients to self-designed questions 1 to 3 regarding the wearable as a primary input device and its integration into daily routines aligned with the usability and data completeness results, affirming our design decisions. In addition, responses to questions 4 and 5 indicated a strong willingness among patients to share patient-generated data, acknowledging the benefits of an RPM system with feedback mechanisms. This reflects a positive attitude from patients toward using technology for chronic disease management, which is consistent with the evaluations by Turner et al [75] and Rising et al [5].

While sensors for cardiovascular monitoring and smartphone apps for PROM recording are advanced, RPM systems are not commonly used in cardiology research or standardized clinical workflows, highlighting the need for more guidance on the design and implementation of such systems. In a systematic review, Kinast et al [76] found a limited number of studies (between January 1, 2001, and March 31, 2021) implementing wearables and noninvasive sensors, as in the activeDCM study, for the management of cardiovascular diseases. In contrast, activeDCM featured continuous recording and transmission of patient-generated data for at least 12 months—more than twice the duration of most studies in the review. This extended time frame offers initial evidence supporting the feasibility of an RPM system for the longitudinal monitoring of patients with chronic diseases. A systematic review by Vegesna et al [11] examining RPM systems for various other chronic diseases, including cardiac, respiratory, neurological, and metabolic conditions, revealed similar distinctions. Only a few studies for each disease type could be identified, shorter monitoring time frames were mainly applied, and the use of wearables as primary device was rather modest (computerized systems, smartphones, websites, biosensors, or a combination of these were often used). Finally, many studies in both aforementioned reviews predominantly emphasized clinical outcomes, with limited attention given to providing in-depth insights into the technical design and implementation of the RPM system as well as its usability, which in turn impacts patient adherence and completeness of patient-generated data—all factors influencing the sustained long-term success of RPM systems [77].

### Limitations

This study has several potential limitations. First, the evaluated cohort consisted of twice as many men than women, which is typical in cardiovascular trials and especially in DCM trials as men are more often affected than women and the activeDCM study design enrolled independently of gender selection. The RPM system can be used in its current implementation state only with iOS devices and requires a Wi-Fi or cellular data connection of the smartphone for data transfers.

Furthermore, the RPM system was only used and evaluated in a research context. We hypothesize that the experience and evaluation results obtained from this research can facilitate the transition of the RPM system into primary care. We anticipate that this transition will maintain high levels of interaction frequency and completeness of patient-generated data, as observed in the research setting. Nevertheless, additional investigation is required to ensure the successful integration and adoption of the RPM system into standardized clinical workflows. Potential challenges, especially in managing a considerably larger number of patients and their devices, may necessitate adjustments to current physician-patient interaction workflows as established for the activeDCM study. Who would bear the costs for the RPM-based treatment approach would also have to be clarified. One possibility for a reimbursement scheme to explore in Germany would be to certify the RPM system as a digital health application [78,79]. As a result, the wearable and smartphone app and the monitoring process could be prescribed by physicians and, thus, paid for by health insurance companies. In addition, cardiologists would need training in interpreting patient-generated data. The calculation of scores and the implementation of clinical decision support tools would streamline the analysis of patient-generated data, thereby saving time and increasing the scalability of RPM-based clinical workflows.

The RPM system module to answer PROM questionnaires has limited response options, using *yes* or *no*, or a scale from 1 to 4. Future efforts should explore methods for adapting more complex PROM questionnaires with diverse response options for use on wearables, maintaining their role as primary input devices. An example could be the Kansas City Cardiomyopathy Questionnaire–Short Version [80], an important questionnaire in cardiology that may require adaptation for wearable use.

Finally, the RPM system’s reliance on manually triggered data transmission by patients, coupled with the consequent time delay in analysis by study physicians, makes it unsuitable for monitoring acute medical emergencies such as myocardial infarctions. To enable real-time monitoring, algorithms would need to be developed for direct analysis of patient-generated data on wearables. Nevertheless, the observational data collected as part of the activeDCM study could serve as a foundation for the future development of such algorithms.

### Conclusions

As life expectancy increases and the older adult population experiences more chronic diseases, there is a growing need for ready-to-use RPM systems. In addition, physicians and researchers require more guidance on designing and implementing such systems for both primary care settings and research purposes [77]. This paper presents a blueprint of an RPM system concept, its subsequent implementation, and its procedure for use as part of the activeDCM study. The selected patient-centric approach and the evaluation results show the feasibility of creating a user-friendly, reusable, interoperable, and secure RPM system including an asynchronous feedback mechanism using a consumer wearable without a designated medical purpose as the primary input device for patient-generated data such as SHD and PROMs. The main factors influencing usability and patient acceptance of the RPM system were device experience, age, and intervention group membership. Our research provides a first step toward integrating RPM systems into standardized clinical workflows and could help other researchers in tailoring RPM systems for their studies. Further research is needed to translate such systems into primary care settings. If this translation is successful, the RPM systems have the potential to change the traditional patient-physician interaction in the future [81].

## Appendix

Multimedia Appendix 1 Paper-based evaluation questionnaire in German.

Multimedia Appendix 2 Screencast showing the functionality of the data viewer with the German user interface.

Multimedia Appendix 3 Health Level 7 Fast Healthcare Interoperability Resources profiles and concrete resource examples of the data model.

Multimedia Appendix 4 CONSORT-EHEALTH (Consolidated Standards of Reporting Trials of Electronic and Mobile Health Applications and Online Telehealth) checklist (v1.6.1).

## Abbreviations

API: application programming interface
APNs: Apple Push Notification service
CG: control group
DCM: dilated cardiomyopathy
ECG: electrocardiogram
Exp–: without previous device experience
Exp+: with previous device experience
FHIR: Fast Healthcare Interoperability Resources
HL7: Health Level 7
IG–: intervention group without feedback messages
IG+: intervention group with feedback messages
JWT: JSON Web Token
LOINC: Logical Observation Identifiers Names and Codes
PROM: patient-reported outcome measure
RPM: remote patient monitoring
SHD: sensor-based health data
SUS: System Usability Scale
UMLS: Unified Medical Language System
