# Supplementary material for: A Remote Patient Monitoring System With Feedback Mechanisms Using a Smartwatch: Concept, Implementation, and Evaluation Based on the activeDCM Randomized Controlled Trial
Source: JMIR Mhealth Uhealth. 2024 Nov 22;12:e58441. doi: 10.2196/58441 (PMC11624455; doi:10.2196/58441)
Supplement: Multimedia Appendix 1 [file mhealth_v12i1e58441_app1.docx]

**Hinweis:** Bitte beziehen Sie sich bei der Beantwortung des Fragebogens auf die neueste Version der App und betrachten Sie die iPhone App (Smartphone) und die Apple Watch App (Wearable) als eine gemeinsame App. Bitte beachten Sie ebenfalls, dass dieser Fragebogen aus 2 Seiten besteht.

1. Ich habe vor der activeDCM Studie schon Erfahrungen mit der Benutzung eines iPhones gemacht:

- Trifft zu
- Trifft nicht zu

1. Ich habe vor der activeDCM Studie schon Erfahrungen mit der Benutzung einer Apple Watch gemacht:

- Trifft zu
- Trifft nicht zu

|  | Stimme gar nicht zu | Stimme nicht zu | Weder noch | Stimme eher zu | Stimme voll zu |
| --- | --- | --- | --- | --- | --- |
| 1. Ich denke, dass ich die activeDCM App häufig benutzen würde. |  |  |  |  |  |
| 1. Ich fand die activeDCM App unnötig komplex. |  |  |  |  |  |
| 1. Ich fand die activeDCM App einfach zu benutzen. |  |  |  |  |  |
| 1. Ich glaube, ich würde die Hilfe einer technisch versierten Person benötigen, um die activeDCM App benutzen zu können. |  |  |  |  |  |
| 1. Ich fand, die verschiedenen Funktionen in der activeDCM App waren gut integriert. |  |  |  |  |  |
| 1. Ich denke, die activeDCM App enthielt zu viele Inkonsistenzen. |  |  |  |  |  |
| 1. Ich kann mir vorstellen, dass die meisten Menschen den Umgang mit der activeDCM App sehr schnell lernen. |  |  |  |  |  |
| 1. Ich fand die activeDCM App sehr umständlich zu nutzen. |  |  |  |  |  |
| 1. Ich fühlte mich bei der Benutzung der activeDCM App sicher. |  |  |  |  |  |
| 1. Ich musste eine Menge lernen, bevor ich anfangen konnte die activeDCM App zu verwenden. |  |  |  |  |  |

|  | Stimme gar nicht zu | Stimme nicht zu | Weder noch | Stimme eher zu | Stimme voll zu |
| --- | --- | --- | --- | --- | --- |
| 1. Die Benutzung der activeDCM App ließ sich gut in meinen Tagesablauf integrieren. |  |  |  |  |  |
| 1. Die Beantwortung des Fragebogens auf der Apple Watch fand ich praktisch. |  |  |  |  |  |
| 1. Die Übertragung meiner Daten (Schritte, Aktivität, Puls, etc.) an die Studienzentrale mittels Apple Watch fand ich praktisch. |  |  |  |  |  |
| 1. Die Übertragung meiner Daten (Schritte, Aktivität, Puls, etc.) an die Studienzentrale mit Rückmeldungen meines Arztes würde ich mir (auch nach der activeDCM Studie) wünschen. |  |  |  |  |  |
| 1. Die activeDCM App mit Rückmeldung meines Arztes ist/wäre nützlich für meine Gesundheit und mein Wohlbefinden. |  |  |  |  |  |
